# Editorial: Small RNAs as a Diverse Toolkit for Bacteria

**DOI:** 10.3389/fmolb.2021.791021

**Published:** 2021-11-10

**Authors:** Olga Ozoline, Konstantin Shavkunov

**Affiliations:** Pushchino Scientific Center for Biological Research of the Russian Academy of Sciences, Laboratory of Functional Genomics and Cellular Stress, Institute of Cell Biophysics of the Russian Academy of Sciences, Pushchino, Russia

**Keywords:** bacterial sRNAs, sRNA processing and function, sRNA-mediated intracellular regulatory pathways, sRNAs for intercellular communication, coding sRNAs

## Introduction

Bacteria use diverse regulatory RNAs to coordinate their physiological processes. Although their spectrum differs from that of higher organisms, the basic principles of action are similar, and many types of non-coding RNAs are present in both kingdoms. Being multifunctional in their ability to interact with various proteins and nucleic acids, small RNAs (sRNAs) encoded by their own genes, as well as fragments of tRNAs, rRNAs, 3′-UTRs, and antisense RNAs, have recently gained increasing attention due to their potential ability to act as signaling molecules establishing interkingdom communications between hosts and microbes. Thus, the goal of the Research Topic was to collect cutting-edge studies exploring biogenesis and RNA-mediated regulatory pathways operating inside bacterial cells and beyond their boundaries.

### Diverse Functions Performed by sRNAs Inside Bacterial Cells

The regulatory potential of sRNAs was addressed in five papers. Focusing on the most recent host-pathogen interaction studies, J. J. González Plaza reviewed sRNA-mediated mechanisms providing “the perspective for pathogens” to survive upon entering a host and withstanding such stresses as temperature and pH shifts, oxidative bursts, and iron and nutrient starvation. In addition to direct interactions of several model sRNAs with target mRNAs, the author also discussed their involvement in the regulation of protein regulators and an ability of RNA chaperones to modulate sRNA activity. Many bacterial pathogens causing infections in metazoans, animals, and plants are discussed in this highly informative review with the conclusion that sRNAs as a therapeutic target provide a great opportunity to tackle antibiotic resistance through gene-based treatment.

The ability of sRNAs to modulate the intracellular concentration of regulatory proteins was evaluated by P. M. Sobrero and Valverde. Focusing primarily on the phylogenetic analysis of CsrA family RNA binding proteins, able to affect transcription termination, translation, and stability for hundreds of target RNAs, the authors also discussed mimic Csr/Rsm family sRNAs that expose multiple short hairpins with an unpaired GGA triplet targeted by CsrA, so that CsrA dimers become sequestered in large nucleoprotein complexes. In *Pseudomonas* pangenome the authors revealed five new subfamilies of Rsm genes and discussed the possibility that multiple paralogues may have evolved for regulation of different subsets of target mRNAs.

London et al. studied participation of sRNAs in the envelope stress response in *E. coli* mediated by σ^E^. The central role in this signal transduction system belongs to the anti-sigma factor RseA, which spans the inner membrane and utilizes its cytoplasmic domain for sequestering σ^E^. By screening a plasmid library with 30 mostly characterized sRNAs, the authors identified RyhB and FnrS as positive regulators of rseA expression at the posttranscriptional level and evidenced a direct interaction between RyhB and RseA-mRNA 5′-UTR.

In their mini review, Quendera et al. observed the main mechanisms employed by sRNAs for regulatory interaction and conducted a comprehensive analysis of information available on the structure of major ribonucleases and RNA-binding proteins (RBPs) that globally mediate sRNA functioning in prokaryotes. Bringing to attention recent evidence for a wide range of functions of these proteins, the authors cover their involvement in sRNA folding, promotion of sRNA/mRNA basepairing *in cis* and *in trans*, and control of sRNA stability. A special focus was placed on the absence of ProQ and/or Hfq homologs in some organisms, indicating that there are more protein partners for sRNAs to be discovered.

Probably the least studied functionality of potentially regulatory RNAs is described in the paper by Ardern et al. The authors investigated short open reading frames identified in RNAs transcribed from the opposite strand of protein coding genes and gave strong evidence for the functional significance of proteins resulting from abundant antisense translation events registered in model bacteria. A demand for the addition of such embedded antisense protein-coding genes to genome annotations and standardization of methods for their prediction is substantiated and novel strategies for research are proposed.

Expression of sRNA-coding genes was addressed in the paper by Kiselev et al. Using a number of available *E. coli* RNA-seq data sets enriched with *in silico* promoter prediction data, the authors analyzed the pattern of transcripts synthesized from divergently transcribed non-coding RNAs. Being subjected to double regulatory pressure and depending on local dynamics of intensively transcribed DNA, those genes often demonstrate substantial heterogeneity in location of apparent transcription start sites. This means that the same locus can produce transcripts differing in length and hence have different folds and stability. Functionality of such isoforms deserves special attention.

### Short RNAs as Signal Molecules in Interkingdom and Interspecies Communications

The paper by Joshi et al. provides the first study characterizing small RNAs released in extracellular vesicles (EV) of an opportunistic pathogen *Staphylococcus aureus*. The authors used iron-depleted medium supplemented with vancomycin, i.e. conditions that mimic an infection treated with antibiotics. As expected, the RNA cargo was enriched with fragments derived from different types of RNAs, but particular attention was paid to the presence of SsrA, RsaC, and RNAIII confirmed by PCR.

**FIGURE 1:**
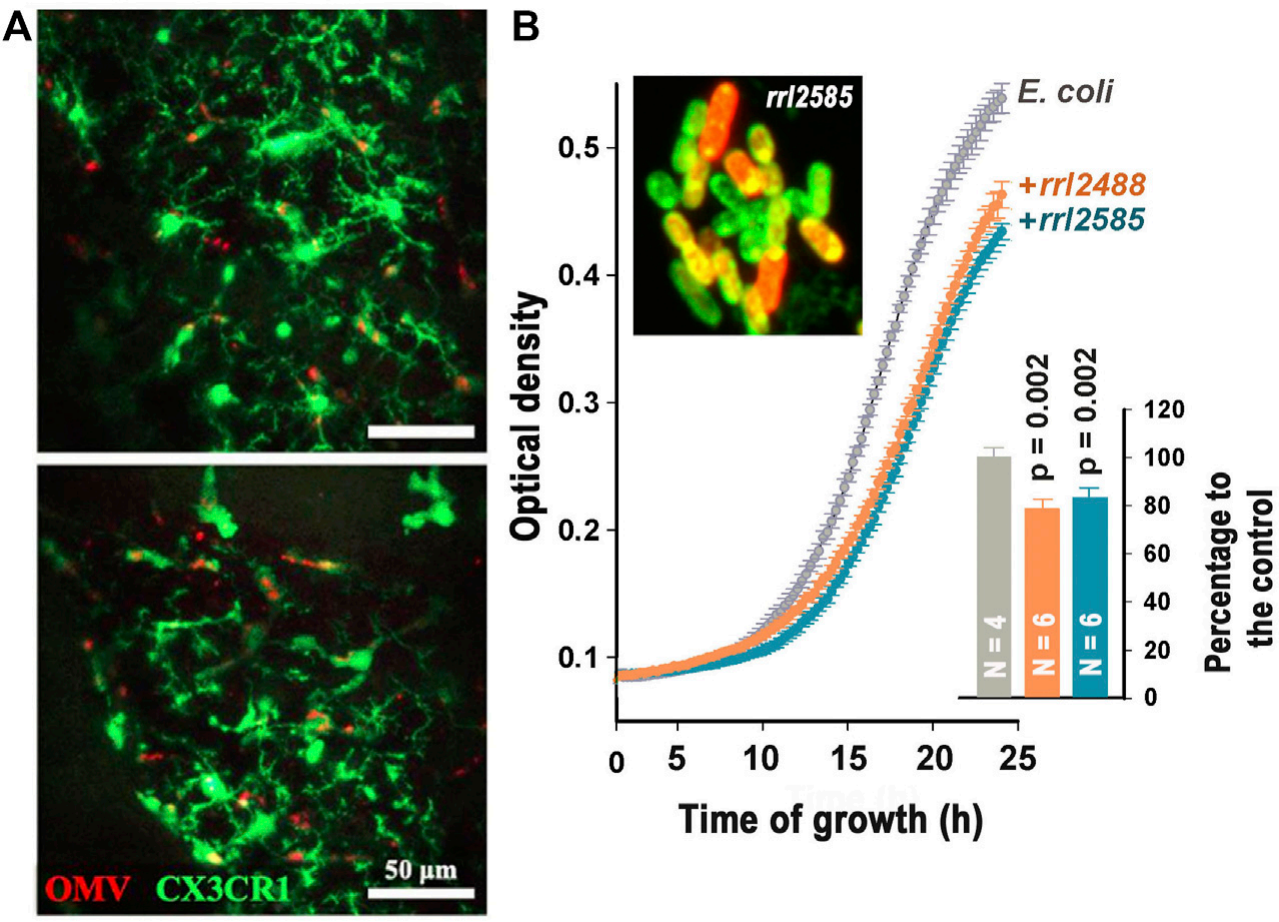
**(A)**: Merged intravital images of murine cortex captured 24 h after injection of *Aggregatibacter actinomycetemcomitans* EVs (OMV) through the tail vein showing colocalization of GFP-stained microglial cells and EVs (red fluorescence). (Ha et al.) **(B)**: Merged image of rrl_2585-Cy 5 (red fluorescence) penetrated into *E. coli* cells and suppression of *E. coli* growth dynamic by two RNA fragments from the secretome of *R. rubrum* (Markelova et al.).

Based on the previous observation that EVs of periodontopathogen *Aggregatibacter actinomycetemcomitans* can cross the blood–brain barrier and that their RNA cargo can promote the secretion of proinflammatory cytokines, Ha et al. tested whether the murine brain immune cells can take up bacterial EVs injected through tail veins and convert this to the immune response. Using intravital imaging of the cortex, the authors for the first time captured injected vesicles in meningeal macrophages and microglial cells (Figure 1A) and confirmed their ability to promote the secretion of proinflammatory cytokines using a murine microglia cell line BV2. Since only RNAse treatment of EVs lysate prevented elevated secretion of IL-6, the authors concluded that RNA rather than DNA cargo is the pivotal factor for immune response.

The effect of EV-RNAs on bladder cells turned out to be different (Dauros-Singorenko et al.). The authors compared the iron-responsive effect of RNA cargo from vesicles released by pathogenic (UPEC 536) and probiotic (Nissle 1917) strains of *Escherichia coli* and found 10-fold more lipopolysaccharides associated with purified RNAs from the pathogenic strain. UPEC EV-RNAs delivered to cultured bladder cells in artificial liposomes changed their transcriptome, while EV-RNAs of a non-pathogenic strain did not. However, the effect of lipopolysaccharides, co-purified with RNA samples, on cytokine secretion and gene expression was almost the same as the effect of UPEC EV-RNAs. Thus, it became clear that the presence of lipopolysaccharides should be taken into account upon analysis of interkingdom communications.

To study interspecies communications, the authors (Markelova et al.) analyzed bacterial RNAs secreted by *Escherichia coli* alone and together with *Prevotella copri* or *Rhodospirillum rubrum*. Differential profiling of reads obtained with RNA-seq revealed the dependence of *E. coli* RNA secretome on the presence of competing bacteria and selecting alien oligonucleotides potentially involved in interspecies communication. An ability of two oligonucleotides to penetrate into *E. coli* cells was tested and confirmed using confocal microscopy. Fragments rrl_2488 and rrl_2585 belonging to *R. rubrum* significantly reduced the growth dynamic of *E. coli* (Figure 1B), while the inhibitory effect of two *P. copri* RNA fragments was observed only in the presence of their complementary oligonucleotides. To our knowledge, this is the first experimental evidence indicating an ability of bacterial RNAs to suppress the growth of other bacteria.

